# Association of Ishii test scores with pneumonia in stable schizophrenic subjects

**DOI:** 10.3389/fpsyt.2022.1034905

**Published:** 2022-10-13

**Authors:** Qin Yang, Sha Huang, Ming Chen, Tian Zhu, Qiuxia Li, Xiaoyan Chen

**Affiliations:** ^1^Psychiatric Hospital of Ziyang, Ziyang, China; ^2^Zigong Affiliated Hospital of Southwest Medical University, Zigong Psychiatric Research Center, Zigong, China

**Keywords:** Ishii test, sarcopenia, schizophrenia, pneumonia, risk

## Abstract

**Aim:**

We investigated the relationship between the sarcopenia-indicating Ishii test scores and pneumonia risk in stable schizophrenia patients.

**Methods:**

This prospective investigation involves schizophrenic inpatients from two mental health centers in western China. Patient baseline information was gathered over 1 month from September 1 to 30 in 2020. All pneumonia-related patient information, including diagnosis and treatment, was acquired over 1 year between October 2020 and October 2021. Patients with schizophrenia were screened for sarcopenia utilizing a threshold value established by Ishii et al. Using regression analysis, the link between Ishii test scores and pneumonia risk in schizophrenia patients was investigated.

**Result:**

This study recruited 232 males and 107 females with schizophrenia over the age of 50 and older. During a 1-year follow-up period, four patients (3 males and 1 female) acquired pneumonia within 1 week of relapse in schizophrenia; therefore, these patients were excluded from the study. Finally, data were collected for 335 patients. The pneumonia incidences were 29.3% in males and 14.2% in females. Our analysis confirmed that compared to the male schizophrenia patients with Ishii test scores < 105 (non-sarcopenia), those with Ishii test scores ≥ 105 (sarcopenia) exhibited an elevated pneumonia risk (OR = 2.739, 95%CI: 1.406–5.333). Following confounders adjustment, Ishii test scores ≥ 105 remained a risk factor for pneumonia (OR = 2.064, 95%CI: 1.029–4.143). Among females with schizophrenia, the Ishii test scores were not associated with pneumonia risk.

**Conclusion:**

In conclusion, our results demonstrated that the Ishii test scores ≥ 105 were strongly associated with pneumonia risk in stable schizophrenic male patients.

## Introduction

Due to schizophrenic patients being hospitalized for long periods, taking antipsychotics, and having a weakened immune system, they have higher risks of other body diseases, including pneumonia (1, 2). A retrospective study showed that the incidence of hospital-acquired pneumonia in patients with schizophrenia aged ≥ 50 years was 7.8% (1). In a Taiwanese study, the incidence of pneumonia in patients with schizophrenia was 1.77 times higher than in patients without schizophrenia (2). Patients with schizophrenia after pneumonia are more likely than those without schizophrenia to have adverse outcomes, such as admission to the intensive care unit, acute respiratory failure, need for mechanical ventilation, and death (2, 3).

Schizophrenic patients usually receive relatively closed treatment in psychiatric hospitals for a long time, and the space is small not to get enough exercise. At the same time, patients take antipsychotic drugs, which have a specific sedative effect; they are often sedentary. Moreover, there is also a lack of professional rehabilitation experts to guide the exercise muscle program. Lack of exercise and being sedentary are risk factors for sarcopenia (4).

Sarcopenia is a progressive and systemic muscle disease (5) that is associated with poor cardiac outcomes (6), respiratory disease (including pneumonia) (7, 8), decreased quality of life (9), and even an increased risk of death (10). Sarcopenia and schizophrenia share some common mechanisms; interleukin (IL)-6 has been implicated in the pathogenesis of sarcopenia (11–13). On the other hand, it has also been implicated in the etiology of schizophrenia due to its ability to cross the blood-brain barrier (14). In addition, human S100B, a small multifunctional protein, is markedly overexpressed in sarcopenia and schizophrenia patients (15). Hence, schizophrenia patients may be more prone to sarcopenia because of specific common mechanisms.

The European Sarcopenia Working Group recommends employing the Ishii test scores for sarcopenia diagnosis (5). The Ishii test scores assess patient age, grip strength, and calf circumference (CC) to identify older patients with sarcopenia. It has a relatively high sensitivity and AUC value (16), with good operability and objectivity, and it is very suitable for institutions without specialized sarcopenia equipment. The Ishii test scores are associated with an increased risk of many clinical adverse outcomes. In a study by Morandi et al. the Ishii test scores were provided to older adults in hospitalized rehabilitation. They revealed that the Ishii test scores-identified sarcopenia patients exhibited an enhanced risk of worse functional status and lower walking ability at discharge (17). Wu et al. also reported that the Ishii test scores-screened sarcopenia strongly correlates with an enhanced risk of cognitive impairment (18). Moreover, the Ishii test scores were successfully employed for the future prediction of undesirable events in patients with heart failure and chronic kidney disease (19, 20). Li et al. demonstrated that the Ishii test scores could predict undesirable outcomes in older patients in clinical practice, including falls, fractures, readmissions, and death (21). Tang et al. also revealed that the Ishii test scores-screened sarcopenia could predict the 3-year mortality of hospitalized older adults (22).

However, no researchers employed the Ishii test scores to estimate the pneumonia risk of schizophrenia patients. In our preliminary study, the Ishii test scores were successfully applied to screen for sarcopenia in schizophrenia patients (4). Therefore, we wanted to examine the association between Ishii test scores and the risk of pneumonia in patients with schizophrenia.

## Materials and methods

### Study design and patient profiles

This study recruited stable schizophrenia inpatients from two mental health centers in Western China to establish a sarcopenia cohort. Baseline patient information was gathered from 1st to 30th September 2020. Additionally, we collected clinical data from pneumonia patients diagnosed and treated at the aforementioned mental health centers between October 2020 and October 2021. Our baseline inclusion and exclusion criteria were based on a previously published article (4). During the follow-up period, cases were eliminated from analysis if pneumonia developed within 1 week of a change in patient status.

This investigation strictly followed the Declaration of Helsinki and was legitimated by the Institutional Review Board (IRB) of mental institutions in western China (IRB number: 20191001; zjsjsbyy-kyxm-2019-2). Before starting the study, informed consent was obtained from each patient or their legal guardians.

### The Ishii test scores

The Ishii test scores were calculated based on age, grip strength, and CC assessments. A digital dynamometer (Camry EH101, El Monte, CA, USA) was employed for patient grip strength measurement. The measurement of CC refers to the measurement methods described in the published articles of our research group (23). All measures were taken by trained personnel from the medical centers where the study was carried out. For the specific calculation method of the Ishii test scores: the male score is calculated as follows: 0.62 × (age – 64) – 3.09 × (grip strength – 50) –4.64 × (CC – 42). The female score is calculated as follows: 0.8 × (age – 64) – 5.09 × (grip strength – 34) – 3.28 × (CC – 42). The diagnostic threshold value of the Ishii test scores followed the recommendations of Ishii et al., and it was as follows: ≥ 105 and ≥ 120 points were the diagnostic cutoffs for male and female sarcopenia, respectively (24).

### Outcome indicator

Pneumonia diagnoses are made by clinicians and treated with medication. Antibiotics are the drugs that must be used. Only patients who suffered from pneumonia for the first time during follow-up were selected for this study. All participants received a chest radiograph or/and computed tomography (CT) scan. Pneumonia was described as an acute lung parenchymal inflammation, combined with acute infiltration on a chest radiograph or CT, along with ≥ 2 of the following symptoms: increased body temperature (≥ 38°C), hypothermia (< 36°C), chills, sweating, novel cough, alteration of discharge color, chest discomfort, or difficulty breathing (25).

### Covariates

Using the hospital electronic system, we identified schizophrenic patients who underwent hospitalization for over 6 months. Next, we collected patient covariate information *via* personal interviews with patients who fit our inclusion criteria. The collected information was as follows: age, sex, weight, height, and smoking and drinking history. Alcohol intake was described as consuming ≥ 150 g ethanol each week (26). Smoking was described as an active smoking history of > 100 cigarettes, and a smoking history included the past 1 year (26). Also assessed were reports of chronic diseases, such as diabetes, hypertension, coronary heart disease (CHD), chronic obstructive pulmonary disease (COPD), and hyperlipidemia, which were treated as additional covariates in this study. Furthermore, Activities of Daily Living (ADL) score (27), depression (PHQ-9 score, Patient Health Questionnaire-9 score) (28), cognitive ability (SPMSQ, The Short Portable Mental Status Questionnaire) (29), antipsychotic (typical antipsychotic and drug kinds), and benzhexol were also assessed as covariates in our analysis. We constructed two models: Model 1: non-adjusted and Model 2: adjusted for covariates like COPD, PHQ-9 in males, COPD, and ADL score in females. This study included a single factor < 0.05 in model 2 for correction. Although there were differences in the distribution of smoking history among females, the frequency of occurrence was too insignificant to be included for correction.

### Statistical analyses

All data analyses employed the SPSS 25.0 software. Two-sided *p*-values < 0.05 were set as the significance threshold. If continuous variables were normally distributed, they were expressed as mean ± SD; otherwise, they were expressed as median (quartile). The baseline characteristics were compared by Student’s *t*-test or Rank-sum, respectively. Data were reported as numbers (percentages) for the statistical description of categorical variables, and Pearson’s chi-square test was employed to compare baseline features. Binomial logistic regression analysis determined the relationship between the Ishii test scores and pneumonia risk in schizophrenia patients.

## Results

**FIGURE 1:**
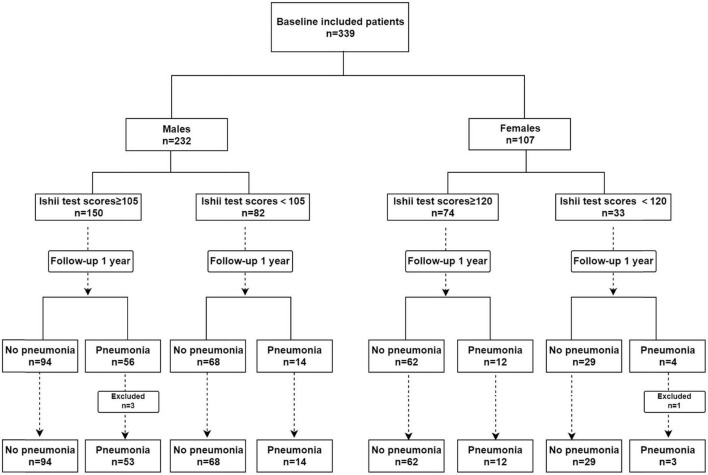
The study profile included patient selection and pneumonia information.

In total, 339 schizophrenia patients aged 50 years and older were enrolled in this study. There were 232 males and 107 females among them. During the 1-year follow-up period, four patients (three males and one female) developed pneumonia within 1 week of schizophrenia relapse; hence, they were eliminated from the analysis. Ultimately, we collected data from 335 patients (Figure 1). The median age of the patients we included was 65, the minimum age was 52, and the maximum age was 81. The incidence of pneumonia was 29.3% among males and 14.2% among females. Among the schizophrenic males, the differences in COPD and the PHQ-9 scores between pneumonia and non-pneumonia cohorts were statistically significant. Among the schizophrenic females, the smoking history, COPD, and ADL scores were statistically significant among pneumonia and non-pneumonia patients (Table 1).

**TABLE 1:** Characteristics of the participants.

General characteristics	Male			Female		
	Non-pneumonia (*n = 162*)	Pneumonia (*n* = 67)	*P*	Non-pneumonia (*n* = 91)	Pneumonia (*n* = 15)	*P*
**Age, year, median (IQR)**	65 (58, 69)	65 (59, 70)	0.307	65 (60, 69)	66 (61, 73)	0.131
**Education, *n* (%)**			0.943			0.08
Primary school and below	112 (70.89)	46 (29.11)		58 (81.69)	13 (18.31)	
Junior high school and above	50 (70.42)	21 (29.58)		33 (94.29)	2 (5.71)	
**Marital status, *n* (%)**			0.128			0.91
Married	36 (80)	9 (20)		35 (85.37)	6 (14.63)	
Unmarried, widowed, divorced	126 (68.48)	58 (31.52)		56 (86.15)	9 (13.85)	
**Smoking history, *n* (%)**			0.573			0.013
No	96 (72.18)	37 (27.82)		91 (86.67)	14 (13.33)	
Yes	66 (68.75)	30 (31.25)		0	1 (100%)	
**Drinking history, *n* (%)**			0.653			–
No	130 (71.43)	52 (28.57)		91 (85.85)	15 (14.15)	
Yes	32 (68.09)	15 (31.91)		0	0	
**Hypertension, *n* (%)**			0.451			0.451
No	126 (72)	49 (28)		69 (87.34)	10 (12.66)	
Yes	36 (66.67)	18 (33.33)		2 2 (81.48)	5 (18.52)	
**Diabetes, *n* (%)**			0.914			0.106
No	132 (70.59)	55 (29.41)		72 (88.89)	9 (11.11)	
Yes	30 (71.43)	12 (28.57)		19 (76)	6 (24)	
**Hyperlipidemia, *n* (%)**			0.749			0.36
No	145 (71.08)	59 (28.92)		85 (86.73)	13 (13.27)	
Yes	17 (68)	8 (32)		6 (75)	2 (25)	
**COPD, *n* (%)**			0.001			0.003
No	132 (76.74)	40 (23.26)		89 (88.12)	12 (11.88)	
Yes	30 (52.63)	27 (47.37)		2 (40)	3 (60)	
**CHD, n (%)**			0.645			0.089
No	158 (70.54)	66 (29.46)		88 (87.13)	13 (12.87)	
Yes	4 (80)	1 (20)		3 (60)	2 (40)	
**ADL score, median(IQR)**	100 (93.75, 100)	100 (90, 100)	0.158	95 (90, 100)	80 (65, 100)	0.041
**PHQ-9 score, *n* (%)**			0.002			0.441
≤ 4	121 (77.07)	36 (22.93)		58 (87.88)	8 (12.12)	
> 4	41 (56.94)	31 (43.06)		33 (82.5)	7 (17.5)	
**SPMSQ score, median (IQR)**	3 (2, 5)	4 (2, 6)	0.077	4 (2, 6)	4 (3, 10)	0.087
**BMI, kg/m** ^2^ **, *n* (%)**			0.174			0.126
** < 18.5 or ≥ 24**	105 (73.94)	37 (26.06)		54 (81.82)	12 (18.18)	
**18.5–24**	57 (65.52)	30 (34.48)		37 (92.5)	3 (7.5)	
**Typical antipsychotic, *n* (%)**			0.968			0.352
No	157 (70.72)	65 (29.28)		86 (85.15)	15 (14.85)	
Yes	5 (71.43)	2 (28.57)		5 (100)	0	
**Antipsychotic, *n* (%)**			0.166			0.692
Alone	140 (72.54)	53 (27.46)		82 (85.42)	14 (14.58)	
Combine	22 (61.11)	14 (38.89)		9 (90)	1 (10)	
**Benzhexol, *n* (%)**			0.116			0.144
No	117 (68.02)	55 (31.98)		62 (82.67)	13 (17.33)	
Yes	45 (78.95)	12 (21.05)		29 (93.55)	2 (6.45)	

COPD, chronic obstructive pulmonary disease; CHD, coronary heart disease; ADL, activities of daily living; PHQ, Patient Health Questionnaire; SPMSQ, Short Portable Mental Status Questionnaire; BMI, body-mass index. For continuous variables, if they were abnormally distributed, the baseline characteristics were compared by Rank-sum.

For categorical variables, Pearson’s chi-square test was employed for comparison of baseline features.

**FIGURE 2:**
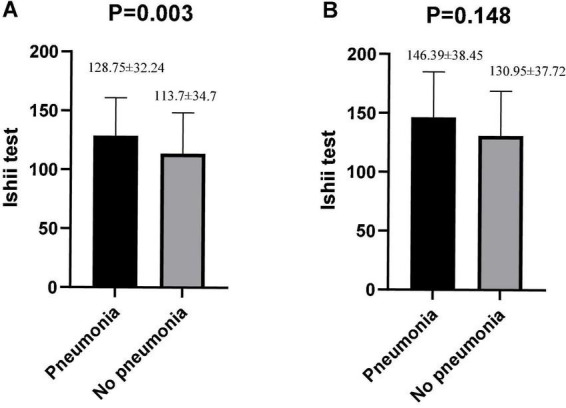
Differences in Ishii test scores between pneumonia and non-pneumonia in males and females. **(A)** Males. **(B)** Females. The Student’s *t*-test was employed for comparison of two groups.

Among the schizophrenic males, patients with pneumonia exhibited a high Ishii test score relative to those without pneumonia (*P* = 0.003, Figure 2). There was no statistical significance among schizophrenic females (*P* = 0.148, Figure 2). The schizophrenic males, who were screened for sarcopenia *via* the Ishii test scores, experienced a higher incidence of pneumonia than those without sarcopenia (sarcopenia vs. non-sarcopenia, 36.05% vs. 17.07%, *P* = 0.002). Interestingly, the schizophrenic females showed no such statistical significance (Table 2).

**TABLE 2:** Analysis of Ishii test scores and incidence of pneumonia.

Variable	Male (*n* = 229)			Female (*n* = 106)		
	No pneumonia *(n* = 162)	Pneumonia (*n* = 67)	*P*	No pneumonia (*n* = 91)	Pneumonia (*n* = 15)	*P*
			0.002			0.354
Ishii test < 105 (M) or Ishii test < 120 (F), *n* (%)	68 (82.93)	14 (17.07)		29 (90.63)	3 (9.37)	
Ishii test ≥ 105 (M) or Ishii test ≥ 120 (F), *n* (%)	94 (63.95)	53 (36.05)		62 (83.78)	12 (16.22)	

M, male; F, female.

Pearson’s chi-square test was employed for comparison of two groups.

We observed that compared to the male schizophrenic patients with Ishii test scores < 105 (non-sarcopenia), those with Ishii test scores ≥ 105 (sarcopenia) experienced a higher risk of pneumonia (OR = 2.739, 95%CI: 1.406–5.333). We adjusted the model for specific confounders, and an Ishii test score ≥ 105 remained a risk factor for pneumonia (OR = 2.064, 95%CI: 1.029–4.143; Table 3). Among females with schizophrenia, the Ishii test scores were not associated with pneumonia risk (Table 3).

**TABLE 3:** Association between Ishii test scores and pneumonia.

Variable	Model 1		Model 2	
	*P*-value	OR (95% *CI*)	*P*-value	OR (95% *CI*)
**Male**				
Ishii test*	0.003	1.013 (1.004–1.022)	0.053	1.009 (1–1.019)
Ishii test < 105	–	1	–	1
Ishii test ≥ 105	0.003	2.739 (1.406–5.333)	0.041	2.064 (1.029–4.143)
**Female**				
Ishii test*	0.148	1.012 (0.996–1.03)	0.942	0.999 (0.981–1.018)
Ishii test < 120	–	1	–	1
Ishii test ≥ 120	0.359	1.871 (0.49–7.144)	0.804	0.827 (0.184–3.725)

*Continuous variable.

Model 1: a non-adjusted model. Model 2: adjusting for COPD, PHQ-9, in males; adjusting for COPD, ADL score in females.

## Discussion

In our study, Ishii test scores were linked to pneumonia in males with schizophrenia, but not in females. This is the first study to use the Ishii test scores to predict pneumonia in schizophrenic patients. In this study, physicians used a simple, accurate sarcopenia screening tool to determine the risk of pneumonia among stable male schizophrenia patients. The advantages of using the Ishii test scores results in clinical practice include their accessibility, accuracy, low cost, and non-invasive aspect. This is a useful platform for psychiatric hospitals that lack specialized equipment.

In our study, the Ishii test scores ≥ 105 were considered sarcopenia and were a risk factor for pneumonia in male patients with schizophrenia. The possible mechanisms are as follows: first, we included people 50 years of age and older who had schizophrenia and had decreased immunity (1). If they with sarcopenia, it will aggravate the decline of immune function (30–32). There is a common molecular mechanism (IL-6) between pneumonia and sarcopenia. IL-6 is secreted during infection or tissue damage (33). As a pro-inflammatory molecule, IL-6 enhances T cell recruitment and expansion and stimulates B cells to produce antibodies (33). Wussler et al. also demonstrated that IL-6 levels in emergency department patients with pneumonia were significantly higher than in non-pneumonic patients (34). On the other hand, IL-6 is a component of age-related chronic low-grade inflammation. In addition, IL-6 attenuates muscle anabolism, energy homeostasis, and even directly mediates muscle catabolism, promotes muscle atrophy, and leads to sarcopenia disease (11–13). Second, schizophrenia patients take long-term antipsychotic drugs, which have a certain sedative effect, the patient’s cough reflex is relatively weak; if there were sarcopenia patients, their respiratory muscle strength will be weakened and cannot effectively regulate cough to clear the airway and more prone to pneumonia (7). Third, sarcopenia also can impair swallowing functions, which leads to aspiration pneumonia (7).

In our study, however, sarcopenia only predicted pneumonia risk in schizophrenic males but not females. There may be differences in the development of sarcopenia between males and females due to differences in sex hormones (35). Absolute and relative reductions in muscle mass were greater in aged males (35). Second, the loss of muscle strength in males was greater and faster, and the decline in physical function was more pronounced (35).

The current study has certain limitations. First, we did not collect information on the pneumonia severity, nor did we gather sufficient mortality data to investigate further the association between the Ishii test scores and pneumonia severity or death. However, follow-up studies are continuing, potentially addressing this issue in the future. Second, the cohort size of this study was relatively small and included only a few schizophrenic patients from western China. Therefore, the results cannot be generalized to other ethnicities and countries; hence, a larger patient population is strongly needed for further validation. Third, there are other suggested cutoffs for the Ishii test’s scores (36). However, in this study, other cutoff values were not explored. Finally, we only included schizophrenic patients aged 50 years and older but did not explore the relationship between the Ishii test scores and pneumonia risk in young adults with schizophrenia.

## Conclusion

This investigation demonstrated that the Ishii test scores ≥ 105 were strongly related to an enhanced pneumonia risk in stable male schizophrenic patients but not females. Therefore, we recommend the application of the Ishii test scores as a reliable screening marker for pneumonia risk among male stable schizophrenia patients.

## Data availability statement

The original contributions presented in this study are included in the article/supplementary materials, further inquiries can be directed to the corresponding author/s.

## Ethics statement

The studies involving human participants were reviewed and approved by the Institutional Review Board of the Zigong Medical Foundation, the Psychiatric Hospital of Ziyang. The patients/participants provided their written informed consent to participate in this study.

## Author contributions

QY, SH, MC, TZ, QL, and XC: study concept and design. QY, TZ, MC, and QL: acquisition of data. XC and SH: analysis and interpretation of data. SH and QY: drafting of the manuscript. XC: critical revision of the manuscript. All authors contributed to the article and approved the submitted version.

## Abbreviations

CC: calf circumference
IRB: Institutional Review Board
CT: computed tomography
CHD: coronary heart disease
COPD: chronic obstructive pulmonary disease
ADL: Activities of Daily Living
PHQ-9: Patient Health Questionnaire-9
SPMSQ: The Short Portable Mental Status Questionnaire
IL: interleukin.
